# Comparison of the Results of Manual and Automated Processes of Cross-Mapping Between Nursing Terms: Quantitative Study

**DOI:** 10.2196/18501

**Published:** 2020-06-09

**Authors:** Fernanda Broering Gomes Torres, Denilsen Carvalho Gomes, Adriano Akira Ferreira Hino, Claudia Moro, Marcia Regina Cubas

**Affiliations:** 1 Graduate Program in Health Technology Pontificia Universidade Católica do Paraná Curitiba Brazil

**Keywords:** health information interoperability, nursing informatics, controlled vocabulary, standardized nursing terminology, ehealth

## Abstract

**Background:**

Cross-mapping establishes equivalence between terms from different terminology systems, which is useful for interoperability, updated terminological versions, and reuse of terms. Due to the number of terms to be mapped, this work can be extensive, tedious, and thorough, and it is susceptible to errors; this can be minimized by automated processes, which use computational tools.

**Objective:**

The aim of this study was to compare the results of manual and automated term mapping processes.

**Methods:**

In this descriptive, quantitative study, we used the results of two mapping processes as an empirical basis: manual, which used 2638 terms of nurses’ records from a university hospital in southern Brazil and the International Classification for Nursing Practice (ICNP); and automated, which used the same university hospital terms and the primitive terms of the ICNP through MappICNP, an algorithm based on rules of natural language processing. The two processes were compared via equality and exclusivity assessments of new terms of the automated process and of candidate terms.

**Results:**

The automated process mapped 569/2638 (21.56%) of the source bank’s terms as identical, and the manual process mapped 650/2638 (24.63%) as identical. Regarding new terms, the automated process mapped 1031/2638 (39.08%) of the source bank’s terms as new, while the manual process mapped 1251 (47.42%). In particular, manual mapping identified 101/2638 (3.82%) terms as identical and 429 (16.26%) as new, whereas the automated process identified 20 (0.75%) terms as identical and 209 (7.92%) as new. Of the 209 terms mapped as new by the automated process, it was possible to establish an equivalence with ICNP terms in 48 (23.0%) cases. An analysis of the candidate terms offered by the automated process to the 429 new terms mapped exclusively by the manual process resulted in 100 (23.3%) candidates that had a semantic relationship with the source term.

**Conclusions:**

The automated and manual processes map identical and new terms in similar ways and can be considered complementary. Direct identification of identical terms and the offering of candidate terms through the automated process facilitate and enhance the results of the mapping; confirmation of the precision of the automated mapping requires further analysis by researchers.

## Introduction

Cross-mapping is a process by which equivalence is established between terms from different health record structures [1-3]. Some of the purposes of cross-mapping are interoperability [4], updating terminological versions [5], and the reuse of terms [6].

Cross-mapping is a nursing strategy that verifies the relevance of decisions arising from clinical reasoning [7] and is a stage in the construction of terminological subsets of the International Classification for Nursing Practice (ICNP) [8]. The construction of subsets of the ICNP and the cross-mapping of its terms with other terminologies are supported by the electronic health (eHealth) program of the International Council of Nurses [9]. The equivalence established in the mapping is performed by comparing one document that contains source terms to another that contains target terms. The similarity between the source and target is determined using the equivalence degree scale proposed by the International Organization for Standardization/Technical Report (ISO/TR) 12.300:2016 [1].

The ICNP is an object for mapping that is composed of primitive terms (in a 7-axis model) and precoordinated terms (diagnoses, results, and nursing interventions) represented in the Web Ontology Language [10-14]. It should be noted that by recognizing the use of terminologies for the documentation of the practices of the nursing profession [15], nursing terminologies can be mapped to each other [2,10] and among other terminologies that are not restricted to the profession [13,16].

Among the challenges of cross-mapping is the high number of terms [2,11,14], which implies that cross-mapping is a strenuous, extensive, and tedious process [11,17] that requires time and effort to develop [18]. Despite initiatives for automation [19,20], called “self-combining mapping” [1], this process is mostly performed manually [2,14,21], which is called “human mapping” [1].

A study that compared the validity of the two mapping approaches demonstrated weaknesses in the results obtained without computational support [22]. As a support tool, Metamap is an algorithm that identifies and maps terms in free English text for the Unified Medical Language System (UMLS) [20], a system whose use promotes the comparison of terms from different terminologies using a unique identifier, the Concept Unique Identifier [23]. With regard to automating mappings between ICNP terms and other terminologies, precoordinated terms should be considered in the UMLS in English [24]. Further, because the primitive terms of the ICNP are arranged in a 7-axis model, natural language processing (NLP) algorithms and techniques can support its mapping [25].

A study that examined the interoperability between nursing information systems mapped nursing diagnoses of the Clinical Care Classification, the ICNP, and the North American Nursing Diagnosis Association-International for the *Systematized Nomenclature of Medicine* *Clinical Terms (*SNOMED CT) through the UMLS. Problems were evidenced in the concordance of the mapping of ICNP with other terminologies by UMLS, which implies interoperability failure [26]. Another study in which the UMLS framework was used to assess the automation of mapping from ICNP to SNOMED CT generated candidate terms for mapping, which facilitated the work of specialists [13].

In this context, the hypothesis of the study that is reported in this paper is as follows: mapping automation, through computational algorithms, collaborates with the manual mapping process. The goal of this study is to compare results of manual and automated term mapping processes to verify if the automated method is adequate to support the task of mapping, considering the challenges of the manual cross-term mapping process [25], the possible contribution of automated mapping to nursing terminologies [11,13,14], and the incipience of studies that compare manual and automated mappings.

## Methods

### Mapping Processes

The descriptive, quantitative study that was used as an empirical basis for this paper examines two term mapping processes: manual [14] and automated [27].

The manual mapping process consisted of mapping 2638 terms of nurses’ records from a university hospital in southern Brazil with 2138 primitive terms of the ICNP (2011 version) and 3894 terms of the ICNP (2013 version) [14]. The database used in this paper is called the University Hospital Terms Bank (*Banco de Termos do Hospital Universitário*, BTHU).

The terms of manual mapping (Table 1) were classified as follows: identical, in which the BTHU term was identical to the ICNP term (eg, source term and target term: impaired); similar, in which the BTHU term was similar to the ICNP term (eg, source term: adipose, target term: adipose tissue); present in the definition of another term of the ICNP, in which the source term was found in the definition of another ICNP term (eg, source term: abrasion, target term: wound); and new, for any outcome that did not fit the previous situations.

In the automated process, the 2638 BTHU terms were mapped with the 2401 primitive terms of the ICNP 2017 using a computational tool called MappICNP, which is available for free on the internet [28]. This tool, which was developed in Python version 3.2, uses lexical and semantic methods from NLP 27 [27]. 

The MappICNP process was structured in two phases. The first phase consisted of normalization of terms from the BTHU and from the ICNP (2401 primitive concepts). This normalization was divided into three steps: accentuation and special character removal, lowercasing, and stopword removal. In the second phase, six NLP rules were created to compare the terms. In all rules except the first one, input and ICNP terms were modified to cover all orthographic variant possibilities. For each rule, the comparison between terms was performed using Levenshtein's distance editing algorithm [27].

In the first rule, each input term was compared to all ICNP terms until a term with 100% similarity was identified. If the similarity was between 90% and 99%, the ICNP term was added to a list of candidate terms that can represent the input term. If this rule achieved 100% similarity, the other rules were not executed [27].

Thus, the automated process performed the mapping as follows:

1. Identical term (rule 1): direct mapping between databases by exact coincidence of the term with equal lexical and semantic structures. For example, the source term and the target term are “vein”.
2. Lemmatizer (rule 2): search for the motto, that is, the ideal lexical unit that represents a set of terms. For example, the source term is “abortion,” and the motto of the target term is “to abort”.
3. Stemmer (rule 3): the terms are reduced to their stems, or radicals. For example, the source term is “medicate” and the stemmer of the target term is “medic”.
4. Synonym (rule 4): a synonym for the source term is identified in an online dictionary. For example, the source term is “person” and the target term is “individual”.
5. Restricted term (rule 5): coincidence with a term that has a more restricted meaning than the source term. For example, the source term is “room” and the target term is “operating room”.
6. Comprehensive term (rule 6): a term is identified with a broader meaning than the source term. For example, the source term is “catheterize bladder” and the target term is “catheterize”.

Terms not mapped by any of the rules were considered new. The BTHU terms were mapped by more than one rule, which generated a higher total number of terms than the manual mapping, that is, 2811 terms (Table 2).

The automated mapping generated a rule that provided the mapping of the term, the percentage of similarity between the source and target terms, the numerical code of the term in the ICNP (ICNP code), the term found in the ICNP (ICNP term), the modification of the term carried out by the rule (ICNP mod), the axis of the term mapped within the ICNP 7-axis model (ICNP axis), the version in which the term was first described in the ICNP (ICNP version), and for each BTHU source term, the candidate target terms in the ICNP 2017.

Table 3 lists examples of candidate target terms for the “clinical” source term and respective additional information.

**Table 1:** Number of terms resulting from manual mapping for each classification of terms in the BTHU (N=2638).

Classification of terms in the BTHU^a^	Terms
Identical	650
Similar	622
Present in the definition of another term of the ICNP^b^	448
New	918

^a^BTHU: Banco de Termos do Hospital Universitário.

^b^INCP: International Classification for Nursing Practice.

**Table 2:** Number of BTHU terms resulting from automated mapping using each mapping rule (N=2811).

Mapping rule	BTHU^a^ terms
Rule 1: Identical term	569
Rule 2: Lemmatizer	122
Rule 3: Stemmer	140
Rule 4: Synonym	525
Rule 5: Restricted term	348
Rule 6: Comprehensive term	76
New terms	1031

^a^BTHU: Banco de Termos do Hospital Universitário.

**Table 3:** Information provided by automated mapping for the source term “clinical”.

Rule	Similarity (%)	ICNP^a^ code^b^	ICNP term^c^	ICNP mod^d^	ICNP axis^e^	ICNP version^f^
2	100	10004459	Clinic	Clinical	Location	1
3	100	10004459	Clinic	Clinic	Location	1
4	100	10014522	Physician	Doctor	Means	1
8	100	10013367	Nursing Clinic	Nursing clinic	Location	1
8	100	10014579	Physiotherapy Clinic	Physiotherapy clinic	Location	1
8	100	10012033	Midwifery Clinic	Obstetrics clinic	Location	1
8	100	10005732	Dental Clinic	Dentist’s clinic	Location	1
8	100	10004463	Clinical Pathway	Clinical conduct	Means	1

^a^ICNP: International Classification for Nursing Practice.

^b^ICNP code: term code in the ICNP.

^c^ICNP term: candidate target term.

^d^ICNP mod: modification of the term carried out by the rule.

^e^ICNP axis: axis of the term in the ICNP.

^f^ICNP version: version in which the term was first described in the ICNP.

### Comparison of the Two Processes

The identical and new terms of the two mapping processes were compared in three stages, with manual mapping being considered the standard.

In the first stage, *assessment of equality and exclusivity*, equality was considered when the output of the automated mapping was the same as the output of the manual process. Here, exclusivity occurred when the mapping resulted in a set of terms mapped by only one of the processes.

In the second stage, *evaluation of new terms of the automated process*, possible equivalent terms were sought in the ICNP 2017. The authors analyzed the definition of each new term with the aid of technical and Portuguese dictionaries and searched for an equivalent term in the terminology. The equivalence degree scale proposed by ISO/TR 12.300:2016 was used: value 1: lexical and conceptual equivalence, value 2: equivalence of meaning with synonymy, value 3: source term broader than the target term, and value 4: source term more restricted than the target term [1]. Values 1 and 2 represent equivalence of meaning, and values 3 and 4 represent the hierarchical relationship; value 3 indicates that the source term is a class of the target term, while value 4 indicates that the source term is a subclass of the target term. In some cases, although a relationship of equivalence was identified, it was not possible to assign a value due to a change in the grammatical class of the terms.

In the third stage, *evaluation of candidate terms*, the terms offered by automated mapping to the new terms of manual mapping were analyzed. The analysis was carried out collaboratively between the authors and nurses participating in a Brazilian research group that studies the ICNP. This analysis considered clinical experience of cross-term mapping and knowledge of the terminology used; thus, it met the quality requirements for mapping proposed by ISO/TR 12.300:2016. The results were determined by consensus, and the semantic relationships between the source and target terms were confirmed using Portuguese language dictionaries and the ICNP 2017.

The terms mapped by the lemmatizer (rule 2), stemmer (rule 3), synonymous terms (rule 4), restricted terms (rule 5), and comprehensive terms (rule 6) were not compared to the results of the manual mapping, as the latter process did not categorize the terms in the same way as the automated mapping rules. For example, the source term “openness” was categorized as similar by the manual process and as restricted by the automated process; however, not all similar terms were equivalent to this rule. Thus, this research was limited to analyzing the identical terms (rule 1) and new terms.

## Results

### Equality and Exclusivity Assessments

Regarding equality, the automated process mapped 569/2638 (21.57%) of the BTHU terms as identical to the ICNP terms, and the manual process mapped 650/2638 as identical (24.64%) (Table 4). The agreement between the processes was 84.46%. The automated process erroneously mapped the source terms “hyperkalaemia” and “reference” to the terms “hypercalcaemia” and “preference”, in which the similarities between the source and target were 92% and 90%, respectively. In mapping new terms, the automated process mapped 1031/2638 (39.08%) of the BTHU terms as new and the manual process mapped 1251/2638 (47.42%) as new (Table 4). The agreement between the processes was 65.70%.

Regarding exclusivity, manual mapping mapped 101/2638 (3.83%) terms as identical and 429/2638 (16.26%) as new, whereas automated mapping mapped 20/2638 (0.75%) as identical and 209/2638 (7.92%) as new (Table 4).

**Table 4:** Absolute and relative frequencies of terms mapped manually and automatically as identical and new according to equality and exclusivity (N=2638), n (%).

Mapping process	Equality		Exclusivity	
	Identical	New	Identical	New
Manual	650 (24.64)	1251 (47.42)	101 (3.83)	429 (16.26)
Automated	569 (21.57)	1031 (39.08)	20 (0.75)	209 (7.92)

### Analysis of New Terms of the Automated Process

Of the 209 terms mapped as new by the automated process, it was possible to establish an equivalence with ICNP terms in 48 cases (23.0%). Examples are shown in Table 5; the others are listed in Multimedia Appendix 1.

### Analysis of Candidate Terms

An analysis of the candidate terms offered by the automated process to the 429 new terms mapped exclusively by the manual process resulted in 100 (23.31%) candidates that had a semantic relationship with the source term (for examples, see Table 6).

**Table 5:** Examples of new source terms identified by the automated process, their equivalent terms in the ICNP, and their degrees of equivalence according to ISO/TR 12.300:2016.

New source term	Equivalent term in the ICNP^a^ (ICNP code)	Degree of equivalence
Alcoholism	Alcohol Abuse (10002137)	2
Apron	Lead Gown (10011222)	3
Bedridden	Confined To Bed (10050397)	2
Infuse	Administering Medication (10025444)	3
Mask oxygen	Oxygen Therapy (10013921)	4
Palpation	Palpating (10013997)	Not attributed
Post-surgical period	Postoperative Period (10027242)	1
Sorotherapy	Intravenous Therapy (10010808)	4
Tracheostomized	Tracheostomy (10019933)	Not attributed
Woman/man Nurse	Nurse (10013333)	1

^a^ICNP: International Classification for Nursing Practice.

**Table 6:** Examples of manual mapping results obtained from evaluation by nurses.

Source term	ICNP^a^ code^b^	ICNP term^c^	ICNP axis^d^
Padded	10007740	Feather Bed Cover	Means
Escort	10042609	Accompanying	Action
Adapt	10001741	Adaptation	Focus
Itch	10010934	Itching	Focus
Stump	10002251	Amputation stump region	Location
Back	10003106	Back	Location
Escape	10027407	Elopement	Focus
Evolution	10015789	Progress	Judgment

^a^ICNP: International Classification for Nursing Practice.

^b^ICNP code: term code in the ICNP.

^c^ICNP term: candidate target term.

^d^ICNP axis: axis of the term in the ICNP.

## Discussion

### Principal Findings

Similarities were found between the results of the manual and automated mapping processes in the identification of identical and new terms. A similar result was found in a research study in which it was concluded that mapping through UMLS performed similarly to mapping performed by specialists [17]. This demonstrates that both processes are able to map the presence of equal terms between terminologies and indicate the absence of representation of source terms in the target documents.

One advantage of the automated process is the organization of the results in an output table. This includes the location of the term in the ICNP 7-axis model, its code, and the version in which it was included in the terminology. This facilitates decision-making regarding the choice of the most suitable target term for objective mapping and contributes to the reduction of selection inaccuracies.

The numeric code improves the mapping process, increases its accuracy [18], and reduces the possibility of incorrect mapping in noncoded databases due to typing and spelling errors. This organization of the results can be carried out by the manual process; however, more time and careful detailing are required for the individual allocation of each term.

Some exclusivities identified in the list of identical and new terms by manual mapping can be explained by three situations: a hierarchical relationship, such as the terms “oxygen therapy”, which is a means in the ICNP, and “oxygen mask”, which is a device that is used to provide oxygen therapy; an equivalence relationship of meaning, such as the terms “sorotherapy” and “intravenous (or endovenous) therapy”; and a list of orthographic equivalences, such as the terms “woman/man nurse” and “nurse”. In this work, these three situations were also evidenced in the evaluation of new terms in the automated process that was performed manually by the authors. In the automated process, the establishment of these relationships would depend on the inclusion of new rules, given the complexity of the Portuguese language. Currently, with the evolution of NLP methods, new rules may be incorporated into MappICNP.

For the hierarchical relationships, the ICNP aims to represent the nursing practice and its various specialties worldwide. Due to the breadth of practice, it becomes impossible to include more specific terms in the subclasses of ontology unless such specificities are essential to the priorities established in the terminological subsets. Thus, depending on the purpose of the mapping, hierarchical relationships between a broader term and a more restricted term are allowed.

Regarding the equivalence relations of meaning, for mapping execution, the use of the ISO/TR 12.300:2016 equivalence degree scale is indicated. This standard allows researchers to establish equivalence of meaning (lexical and conceptual), synonymy, scope, and restriction of meaning of the terms [1].

Regarding relationships of orthographic equivalence, source terms from nonstandard bases require normalization [8]. This process, which precedes the mapping, is essential to minimize errors and reduce the number of source terms. The normalization of terms requires caution in relation to the use of traditional rules, among them the substitution of the term female for male. In addition, in this case, the researcher’s knowledge about the target terminology is crucial. A similar situation was indicated in a previous study in which normalization was performed only when pertinent. For example, the term “right”, when appropriate to the male, can refer to the “focus” axis (patient’s right) or the “location” axis (right side) [11].

When the source document consists of nursing records in natural language, the results of the mapping can be affected by the writing of the terms. For example, the source term “tracheostomized”, which was categorized as new by the automated process, had an established equivalence to the term “tracheostomy”. The adjective “tracheostomized” was registered by the nurses to refer to a location that is represented in the ICNP by the noun “tracheostomy” [9].

Although automated mapping considers the lexical and semantic structures of the terms, there is a need for evaluation of the nonexplicit relations by a researcher. An example is found in [29], in which the automated mapping of the term “mood stabilizer” was related to the term “mast cell stabilizer”. This result implies that equivalence errors can occur if there is no expert evaluation of automated mapping results.

In turn, manual mapping is more time-consuming and depends on the experts’ knowledge of the terminology used [18]. In addition, the analysis should provide strategies to minimize precision errors in the selection of the target term, including the use of technical and English dictionaries and structured vocabularies such as the Health Sciences Descriptors (*Descritores em Ciências da Saúde*, DeCS) and Medical Subject Headings.

In this study, the exclusive use of the English dictionary to map synonymous terms was identified as a limitation of MappICNP. This could be seen in the term “acromion”, which was defined in the dictionary as “scapula apophysis, in the form of a spatula” and in the DeCS as “lateral extension of the spine of the scapula and the highest point of the shoulder.” The last definition enables the mapping of “shoulder” as a term candidate in the ICNP.

The percentage of similarity of the candidate term assists the specialists in analyzing equivalence errors; this allows the manual analysis to be directed to the terms whose similarity is not 100%. The automatic offer of candidate terms expands the possibility of choosing target terms and increases the time for selecting alternatives. An example of this was demonstrated by automated mapping of ICNP terms to SNOMED CT, in which the source term “tobacco (or smoke) abuse” generated the candidate terms “tobacco abuse” and “tobacco addiction syndrome” for evaluation by the specialists [13].

In this research, the relevance of the candidate terms could be seen in the nurses’ analysis. One-third of the new terms identified by manual mapping had equivalents, demonstrating that the use of an automated process can minimize weaknesses in the manual process.

Although it was not an objective of this study, the time spent by the automated process was shorter than that spent in the manual process. The schedule of the study in which the manual mapping was performed was 3 months for the mapping stage, while automated mapping processed the rules in less than 12 h. The time optimized in this step through the automated process can be directed to the manual analysis of the candidate terms.

### Limitation of This Study

As a limitation of this study, the use of different versions of ICNP and the exclusive use of the primitive terms of the ICNP in the manual mapping with the 2011 version and in the automated process should be taken into consideration. Due to this limitation, it was impossible to compare potential results in relation to the precoordinated terms. It is expected that the standardization for categorizing mapping results proposed in the ISO/TR 12.300:2016 equivalence grade scale will contribute to overcoming this limitation in future research studies.

### Conclusion

Identical and new terms are similarly mapped by automated and manual processes; hence, it has been concluded that these processes can be complementary. Although the automated process requires manual analysis by a researcher to confirm the accuracy of the terms, it facilitates and enhances the results of mapping by identifying identical terms and candidate terms.

The importance of one process complementing the other is the ability to use different methods of mapping terms so that the result is better than the performance of each process separately.

Given the complexity of hierarchical, equivalence, and orthographic relationships, analysis by specialists is essential to establish equivalences not identified by the automated process. However, with the aid of automation, the time to perform the analysis is reduced.

The results of this research can contribute to improving the MappICNP tool. As a contribution to nursing, these results support the construction of terminology subsets of the ICNP with regard to the cross-mapping stage and can aid the comparison of nursing practices in different scenarios.

An additional contribution of this study is that interdisciplinarity was established to achieve the proposed objective, providing opportunities for the integration of different knowledge from nursing and informatics.

## Appendix

Multimedia Appendix 1 New source terms identified by the automated process, their equivalent terms in the ICNP, and their degrees of equivalence by ISO/TR 12.300:2016.

## Abbreviations

BTHU: Banco de Termos do Hospital Universitário
DeCS: Descritores em Ciências da Saúde
eHealth: electronic health
ICNP: International Classification for Nursing Practice
ISO/TR: International Organization for Standardization/Technical Report
NLP: natural language processing
SNOMED CT: Systematized Nomenclature of Medicine Clinical Terms
UMLS: Unified Medical Language System

## References

[1] International Organization for Standardization (2016). ISO/TR 12300: Health informatics: Principles of mapping between terminological systems.

[2] Ivory CH (2016). Mapping Perinatal Nursing Process Measurement Concepts to Standard Terminologies. Comput Inform Nurs.

[3] Amorim IS, Beckhauser E, Petrolini VA, Savaris A, Wangenheim AV (2018). Mapeamento terminológico no domínio da radiologia obstétrica: estudo estatístico a partir de ontologias. Rev Eletron Comun Inf Inov Saúde.

[4] Diehl AD, Meehan TF, Bradford YM, Brush MH, Dahdul WM, Dougall DS, He Y, Osumi-Sutherland D, Ruttenberg A, Sarntivijai S, Van Slyke CE, Vasilevsky NA, Haendel MA, Blake JA, Mungall CJ (2016). The Cell Ontology 2016: enhanced content, modularization, and ontology interoperability. J Biomed Semant.

[5] Tanno LK, Calderon M, Papadopoulos NG, Demoly P, EAACI/WAO Task force of a Global Classification of Hypersensitivity/Allergic diseases (2015). Mapping hypersensitivity/allergic diseases in the International Classification of Diseases (ICD)-11: cross-linking terms and unmet needs. Clin Transl Allergy.

[6] Kamdar MR, Tudorache T, Musen MA (2017). A Systematic Analysis of Term Reuse and Term Overlap across Biomedical Ontologies. Semant Web.

[7] Morais SCRV, Nóbrega MMLD, Carvalho ECD (2018). Cross-mapping of results and Nursing Interventions: contribution to the practice. Rev Bras Enferm.

[8] Carvalho CMG, Cubas MR, Nóbrega MMLD (2017). Brazilian method for the development terminological subsets of ICNP®: limits and potentialities. Rev Bras Enferm.

[9] International Council of Nurses (2018). International Classification for Nursing Practice (ICNP) version 2017.

[10] Tannure MC, Salgado PDO, Chianca TCM (2014). Cross-Mapping: diagnostic labels formulated according to the ICNP versus diagnosis of NANDA International. Article in Portuguese. Rev Bras Enferm.

[11] Gomes DC, Cubas MR, Pleis LE, Shmeil MAH, Peluci APVD (2016). Terms used by nurses in the documentation of patient progress. Rev Gaucha Enferm.

[12] Coenen A, Hardiker N, Jansen K, Kim TY, International Council of Nurses (2018). ICNP to SNOMED-CT (Systematized Nomenclature of Medicine Clinical Terms) equivalency table for intervention statements: Terminology cross-mapping 2018.

[13] Kim TY (2016). Automating lexical cross-mapping of ICNP to SNOMED CT. Inform Health Soc Care.

[14] Cubas M, Pleis L, Gomes D, Costa E, Peluci A, Shmeil M, Carvalho C (2017). Mapping and definition of terms used by nurses in a hospital specialized in emergency and trauma care. Rev. Enf. Ref.

[15] (2018). American Nurses Association.

[16] Boyd AD, Dunn Lopez K, Lugaresi C, Macieira T, Sousa V, Acharya S, Balasubramanian A, Roussi K, Keenan GM, Lussier YA, Li J', Burton M, Di Eugenio B (2018). Physician nurse care: A new use of UMLS to measure professional contribution: Are we talking about the same patient a new graph matching algorithm?. Int J Med Inform.

[17] Hsiao M, Chen C, Chen J (2009). Using UMLS to construct a generalized hierarchical concept-based dictionary of brain functions for information extraction from the fMRI literature. J Biomed Inform.

[18] Saitwal H, Qing D, Jones S, Bernstam EV, Chute CG, Johnson TR (2012). Cross-terminology mapping challenges: a demonstration using medication terminological systems. J Biomed Inform.

[19] Gu H, Chen Y, He Z, Halper M, Chen L (2016). Quality Assurance of UMLS Semantic Type Assignments Using SNOMED CT Hierarchies. Methods Inf Med.

[20] Aronson AR, Lang F (2010). An overview of MetaMap: historical perspective and recent advances. J Am Med Inform Assoc.

[21] Clares J, Nóbrega M, Guedes M, Silva L, Freitas M (2016). Bank of terms for clinical nursing practice with community elderly. Bank of terms for clinical nursing practice with community elderlyRev Eletr Enf 2016 Jun.

[22] Choi Y, Jung C, Chae Y, Kang M, Kim J, Joung K, Lim J, Cho S, Sung S, Lee E, Kim S (2014). Comparison of validity of mapping between drug indications and ICD-10. Direct and indirect terminology based approaches. Methods Inf Med.

[23] Kim TY, Hardiker N, Coenen A (2014). Inter-terminology mapping of nursing problems. J Biomed Inform.

[24] International Council of Nurses (2011). International Classification for Nursing Practice (ICNP) version 2.0.

[25] Gomes DC, Oliveira LESE, Cubas MR, Barra CM (2019). Use of Computational Tools as Support to the Cross-Mapping Method Between Clinical Terminologies. Texto contexto - enferm.

[26] Kim TY, Coenen A, Hardiker N (2012). Semantic mappings and locality of nursing diagnostic concepts in UMLS. J Biomed Inform.

[27] Ronnau LB, Torres FBG, E Oliveira LES, Gomes DC, Cubas MR, Moro C (2019). Automatic Mapping Between Brazilian Portuguese Clinical Terms and International Classification for Nursing Practice. Stud Health Technol Inform.

[28] GitHub.

[29] Nelson SD, Parker J, Lario R, Winnenburg R, Erlbaum MS, Lincoln MJ, Bodenreider O (2017). Interoperability of Medication Classification Systems: Lessons Learned Mapping Established Pharmacologic Classes (EPCs) to SNOMED CT. Stud Health Technol Inform.

