# Supplementary material for: Comparison of the Results of Manual and Automated Processes of Cross-Mapping Between Nursing Terms: Quantitative Study
Source: JMIR Nurs. 2020 Jun 9;3(1):e18501. doi: 10.2196/18501 (PMC8293700; doi:10.2196/18501)
Supplement: Multimedia Appendix 1 [file nursing_v3i1e18501_app1.docx]

Multimedia Appendix 1 - New source terms by automated process, their equivalent terms in ICNP, and degree of equivalence by ISO/TR 12.300:2016.

| **Source term—new** | **Equivalent term in ICNP** | **Degree of equivalence** |
| --- | --- | --- |
| Alcoholism | Alcohol Abuse (10002137) | 2 |
| Ambulatory | Outpatient Department (10013852) | 2 |
| Anuria | Urinary frequency (10046695), impaired (10012938) | 2 |
| Apron | Lead Gown (10011222) | 3 |
| Bedridden | Confined To Bed (10050397) | 2 |
| Bolus | Infusion Technique (10010189) | 4 |
| Bronchoaspiration | Aspiration (10002656) | 2 |
| Catarrh | Secretory Substance (10017635) | 4 |
| Crust | Ulcer (10020237) | 4 |
| Denture | Denture (10005750) | 2 |
| Diet Therapy | Nutritional Therapy (10013442) | 2 |
| Diuresis | Urine (10020478) | 2 |
| Dysarthria | Slurred Speech (10018304) | 2 |
| Evening | Afternoon (10001955) | 2 |
| Eye drops | Medication (10011866) | 4 |
| Fluid therapy/serotherapy | Intravenous Therapy (10010808) | 4 |
| Hospitalization unit | Health Care Department (10008724) | 4 |
| Inflammatory | Inflammation (10010127) | Not attributed |
| Infuse | Administering Medication (10025444) | 3 |
| Infusion pump | Infusion Device (10033352) | 4 |
| Mask oxygen | Oxygen Therapy (10013921) | 4 |
| Mouth | Oral Cavity (10013720) | 2 |
| Palpation | Palpating (10013997) | Not attributed |
| Pododactyl | Toe (10019797) | 2 |
| Post-surgical period | Postoperative Period (10027242) | 1 |
| Pronation | Prone (10015829) | 2 |
| Prone position | Prone (10015829) | 2 |
| Prophylaxis | Administering Prophylactic Treatment (10001827) | 2 |
| Resuscitation | Cardiopulmonary Resuscitation (10043019) | 2 |
| Removal | Removing (10016763) | Not attributed |
| Runny nose | Secretory Substance (10017635) | 4 |
| Scrotal | Scrotum (10017603) | 1 |
| Secretive | Secretory Substance (10017635) | Not attributed |
| Sepsis | Infection (10010104) | 4 |
| Serotherapy | Intravenous Therapy (10010808) | 4 |
| Supervisor | Supervising (10019093) | Not attributed |
| Supine | Supine (10019103) | 2 |
| Sweating | Perspiration (10014449) | 2 |
| Tachypneic | Respiration Rate (10016904), high (10009007) | 2 |
| Thumb | Finger (10007937) | 4 |
| Tracheostomized | Tracheostomy (10019933) | Not attributed |
| Truck | Vehicle (10020654) | 4 |
| User | Patient (10014132) | 2 |
| Venoclysis | Intravenous Therapy (10010808) | 2 |
| Verbalization/verbalize | Communication (10004705) | 2 |
| Wadding | Wound Dressing (10021227) | 4 |
| Walk | Ambulation Technique (10002222) | 2 |
| Woman/man Nurse | Nurse (10013333) | 1 |
